# Supplementary material for: Integrative Genomics and Bioactivity-Guided Isolation of Novel Antimicrobial Compounds from Streptomyces sp. KN37 in Agricultural Applications
Source: Molecules. 2024 Apr 28;29(9):2040. doi: 10.3390/molecules29092040 (PMC11085741; doi:10.3390/molecules29092040)
Supplement: Supplementary file 1 [file molecules-29-02040-s001.zip › molecules-2949002-supplementary.pdf]

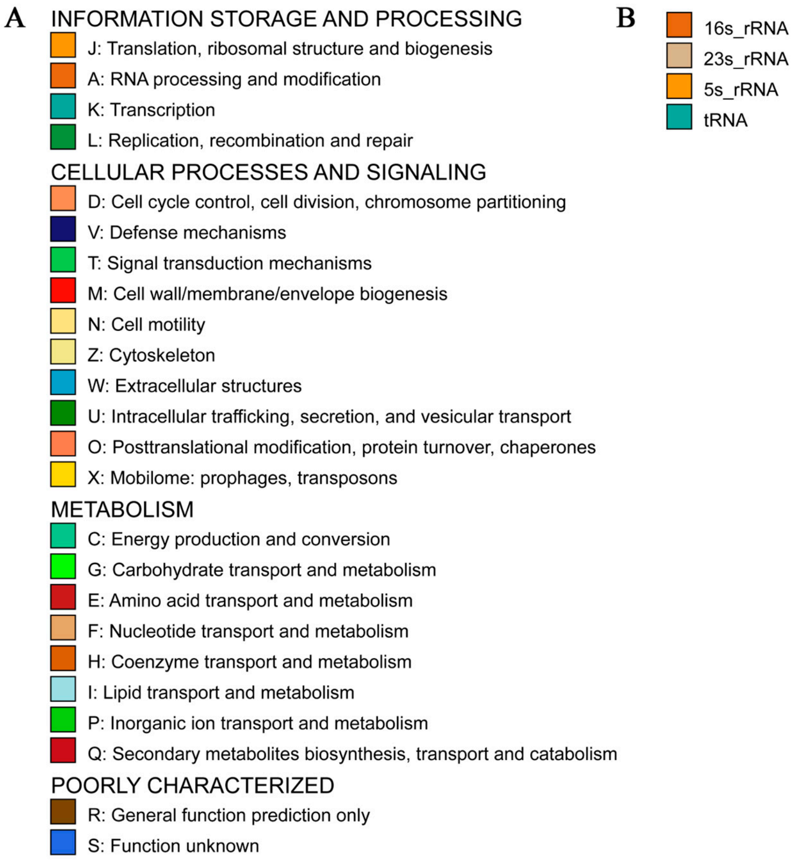

Figure S1 The COG legend (A) and ncRNA legend (B) of outermost color region in whole-genome mapping.

TLC analytical method

A small amount of ethyl acetate crude extract was taken and dissolved in methanol. A total of 2 μL was pipetted into a 0.33 capillary tube and spotted at 1 cm from the lower edge of a GF254 silica gel plate (25 mm × 75 mm). The upright upward unfolding method was used, and when the front edge of the unfolding agent was 1 cm away from the upper edge of the silica gel plate, the silica gel chromatography plate was removed and the position of the front edge of the solvent was marked, and then the results of the unfolding were observed under a UV analyzer.

The crude extracts were unfolded with five organic reagents: petroleum ether, ethyl acetate, dichloromethane, methanol and isopropanol. According to the unfolding conditions, the polarity

range of the crude extracts was initially determined, and then the thin-layer chromatographic unfolding system with different polarities was configured to determine the elution system of the silica gel column chromatography.

When recovery was required, a thicker separating thin-layer chromatographic plate was selected and the silica powder at the target location was carefully scraped off the glass plate with a knife and extracted with ethyl acetate and a small amount of methanol.

Column chromatography separation method

The weight ratio of sample and silica gel is 1:30. So, 30 g of ethyl acetate extract sample was taken, weighing 200–300 mesh silica gel powder of 900 g. In the bottom of the washed chromatography column stuffed with skimmed cotton, quartz sand was laid about 1 cm thick and 500 mL of petroleum ether was added to be used. Moreover, 900 g of silica gel powder was added to 2 L of petroleum ether in a 4 L plastic measuring cup and stirred well with a glass rod.

A clean 2 L beaker was placed at the bottom of the column, a solid sampling funnel was used to pour in the stirred silica gel homogenate at one time, a suction ear ball was used to continuously tap the wall of the column and open the bottom switch, and the pressurized tapping was repeated to make the silica gel column compacted and close the bottom switch. After the silica gel column was thoroughly compacted and the surface was flat, the switch was opened, so that the liquid level in the column was about 2 cm in the upper part of the silica gel column, and then a 1 cm thick layer of quartz sand was added along the wall of the dialysis column tube with a medicine spoon to keep the liquid level in the upper part of the quartz sand at about 1 cm. The formula for calculating the column volume is as follows: Column volume = 500 mL + Homogenization volume - Outflow volume.

Using the wet-loading method, the lower switch of the chromatography column was turned on until the liquid level was close to the quartz sand. Using a dropper, the sample was added evenly to the surface of the quartz sand, trying to keep the liquid level in the column even with the quartz sand. Gradient elution was performed using petroleum ether, ethyl acetate and methanol, using 5–6 column volumes for each elution system.

Semi-preparative high-performance liquid chromatography purification

Chromatographic column: HPLC-5C18E (20 × 250 mm, 5 μm). Flow velocity: 10 mL/min. Sample marking size: 100 μL. Column temperature: 35°C. Detecting wavelength: 254 nm. Flow phase A: methanol (HPLC). Flow phase B: 1% formic acid-deionized water.

These three elution systems were mainly used for separation, and the operation was flexibly adjusted according to the separation effect (**Tables S1–S3**). The collected peaks were dried for biological activity testing to obtain active substances with high purity.

Table S1 Gradient elution conditions of the mobile phase.

**Time (min)**	**A (%)**	**B (%)**	**Flow velocity (mL/min)**
**0**	5	95	10
**2**	15	85	10
**17**	25	75	10
**27**	99	1	10
**35**	99	1	10
**40**	5	95	10
**45**	5	95	10

Table S2 Gradient elution conditions of the mobile phase.

**Time (min)**	**A (%)**	**B (%)**	**Flow velocity (mL/min)**
**0**	5	95	10

**2**	35	65	10
**17**	75	25	10
**27**	99	1	10
**35**	99	1	10
**40**	5	95	10
**45**	5	95	10

Table S3 Gradient elution conditions of the mobile phase.

**Time (min)**	**A (%)**	**B (%)**	**Flow velocity (mL/min)**
**0**	5	95	10
**2**	55	45	10
**17**	85	25	10
**27**	99	1	10
**35**	99	1	10
**40**	5	95	10
**45**	5	95	10

Overview of the separation process

We divided the ethyl acetate crude extract into 9 fractions and then determined their antifungal activity against *Rhizoctonia solani* at a concentration of 500 mg/L (**Figure S2**). Each of these fractions weighs was used: Fr.1 9.3675 g; Fr.2 0.7053 g; Fr.3 14.2212 g; Fr.4 2.5752 g; Fr.5 1.9912 g; Fr.6 1.8751 g; Fr.7 1.0812 g; Fr.8 1.7201 g; Fr.9 2.2504 g. We chose the fractions with stronger bioactivity and more weight for further isolation and finally obtained 8 compounds (**Figure S3**).

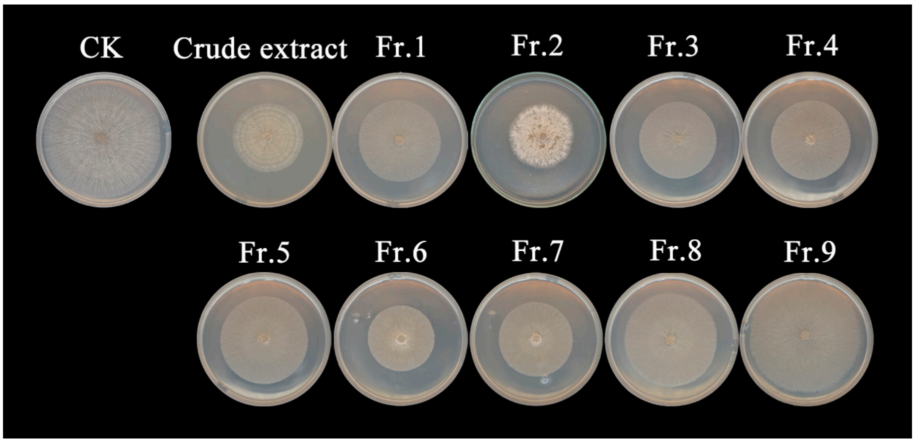

Figure S2 Inhibitory effect of nine fractions at 500 mg/L on *Rhizoctonia solani*.

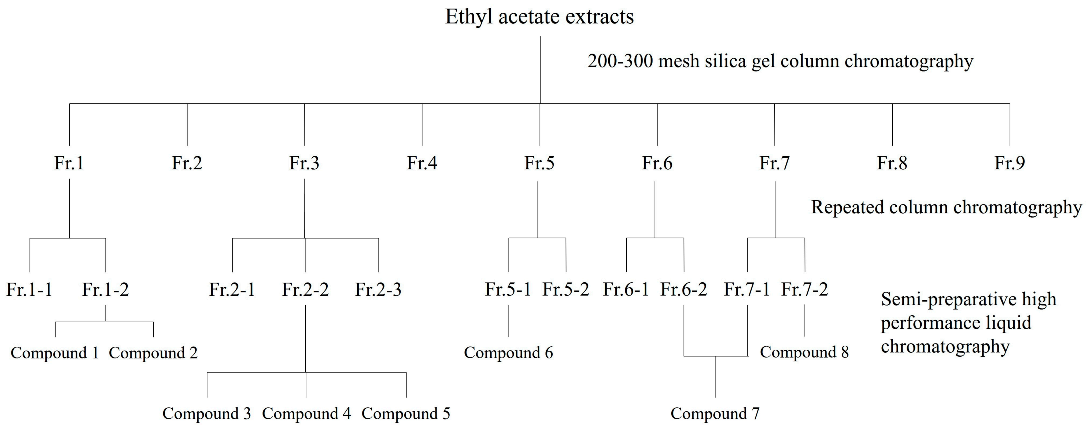

Figure S3 Overview of the separation process.

4-(Diethylamino)salicylaldehyde (**1**): ^{1}H NMR (400 MHz, $CDCl_3$) δ = 11.62 (s, 1H), 9.50 (s, 1H), 7.28 (s, 1H), 6.28 (dd, *J*=8.9, 2.4, 1H), 6.09 (d, *J*=2.4, 1H), 3.41 (q, *J*=7.1, 4H), 1.21 (t, *J*=7.1, 6H). ^{13}C NMR (101 MHz, $CDCl_3$) δ = 192.11, 164.51, 154.20, 135.53, 111.70, 104.67, 97.04, 45.10, 12.69.

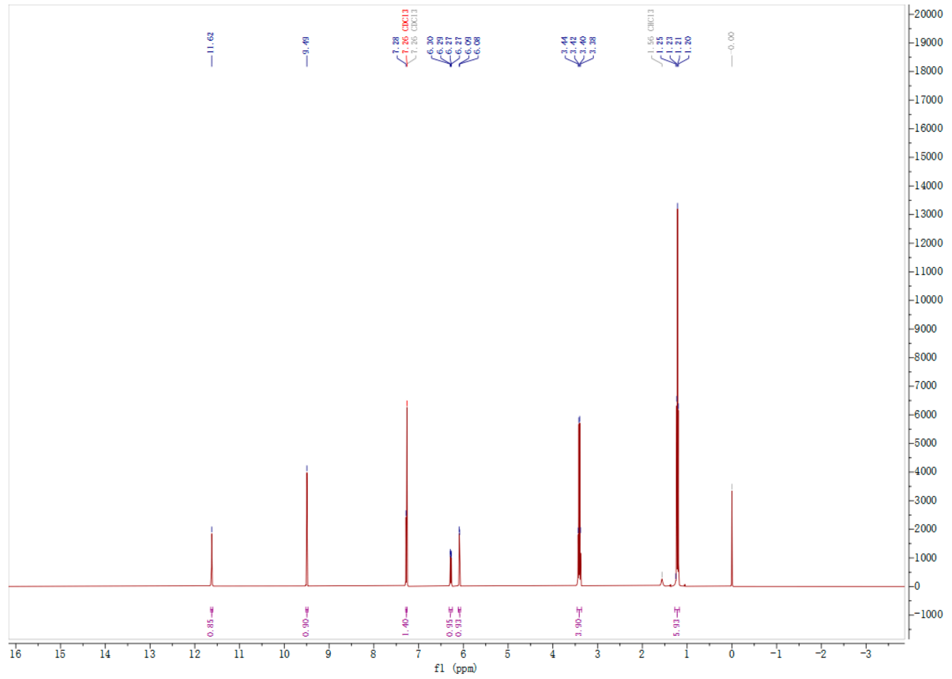

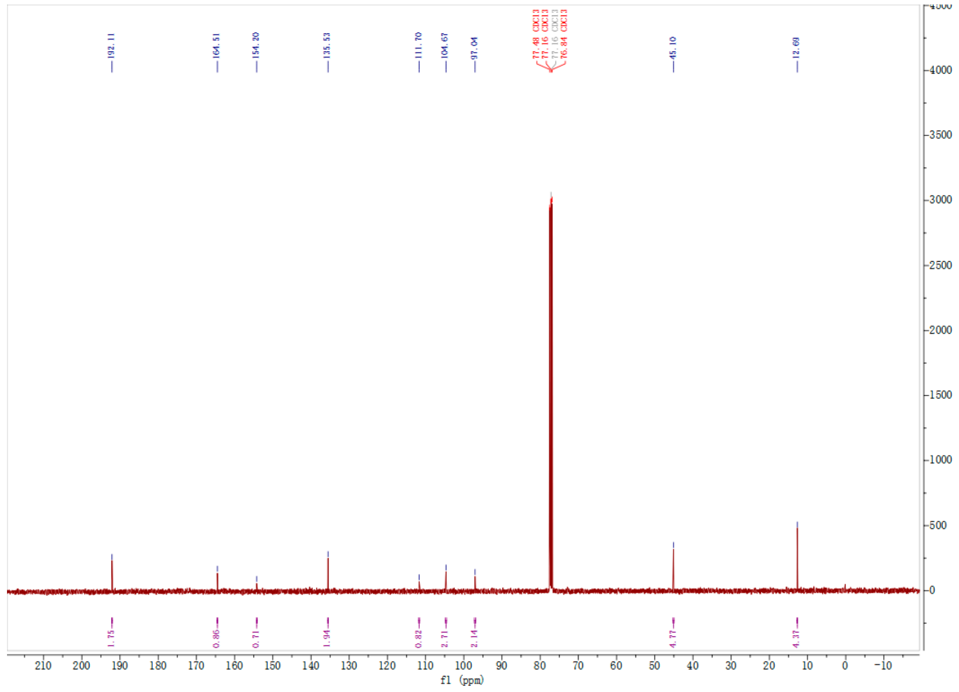

Figure S4 ^{1}H NMR and ^{13}C NMR of 4-(Diethylamino)salicylaldehyde.

4-Nitrosodiphenylamine (2): ^{1}H NMR (400 MHz, $CDCl_3$) δ = 7.94–7.72 (m, 2H), 7.48–7.37 (m, 2H), 7.32–7.17 (m, 2H), 7.05–6.92 (m, 2H). ^{13}C NMR (101 MHz, $CDCl_3$) δ = 164.13, 151.80, 139.01, 129.93, 125.48, 122.85, 113.56.

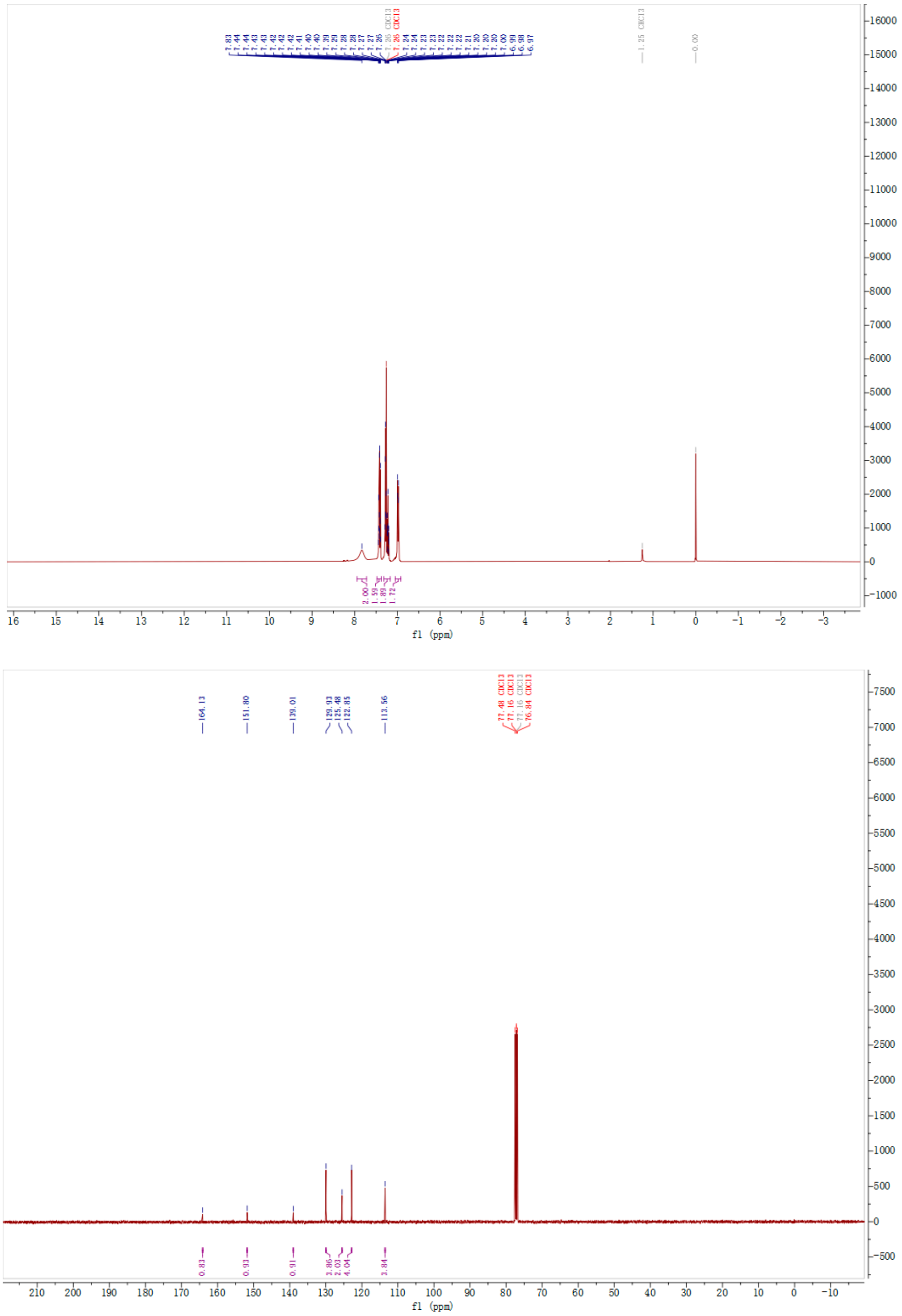

Figure S5 ^{1}H NMR and ^{13}C NMR of 4-Nitrosodiphenylamine.

N-(2,4-Dimethylphenyl)formamide (3): ^{1}H NMR (400 MHz, CDCl3) δ = 8.51–8.38 (m, 1H), 7.09–6.59 (m, 3H), 2.30 (d, J=5.7, 3H), 2.25 (d, J=4.3, 3H). ^{13}C NMR (101 MHz, $CDCl_3$) δ = 163.40, 136.13, 135.50, 132.02, 127.75, 127.50, 121.33, 21.01, 17.81.

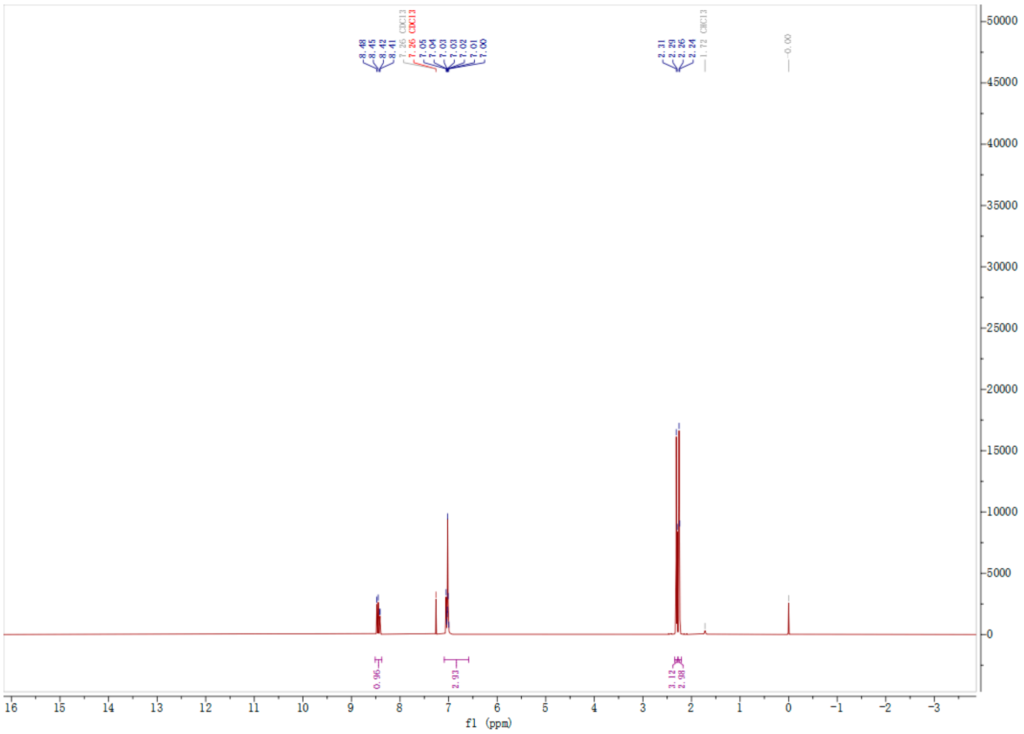

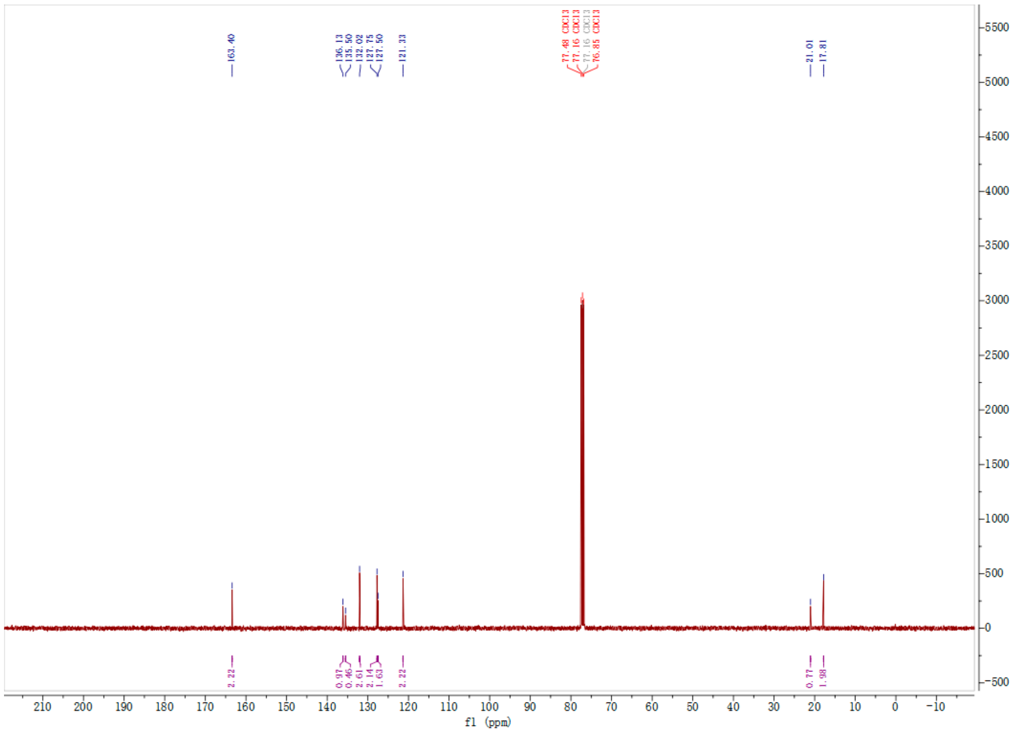

Figure S6 ^{1}H NMR and ^{13}C NMR of N-(2,4-Dimethylphenyl)formamide.

4-Nitrocatechol (**4**): ^{1}H NMR (400 MHz, DMSO) δ = 10.51 (s, 1H), 10.04 (s, 1H), 7.67–7.56 (m, 2H), 6.89 (d, *J*=8.7, 1H). ^{13}C NMR (101 MHz, DMSO) δ = 153.33, 145.90, 139.92, 116.97, 115.46, 110.84.

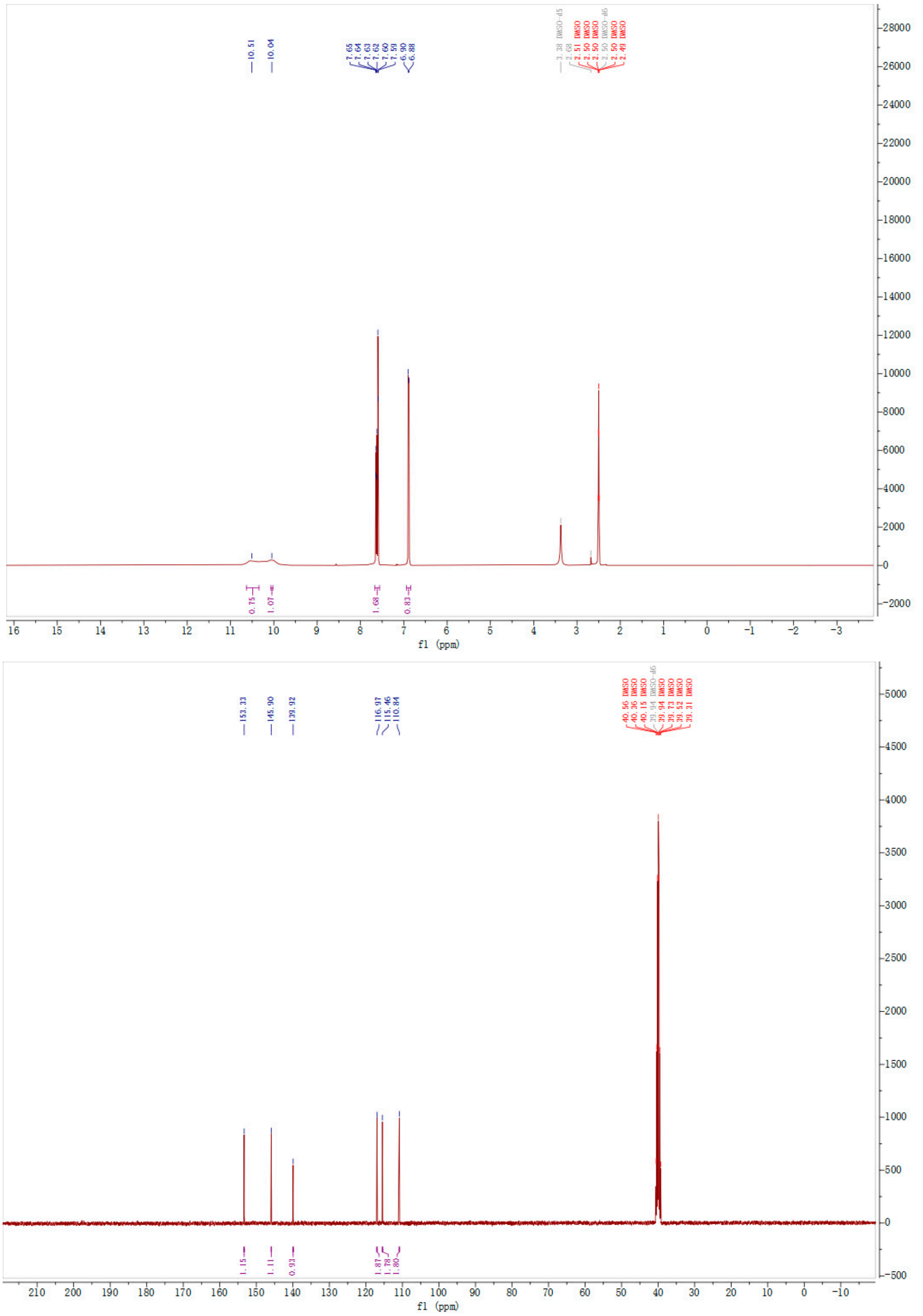

Figure S7 ^{1}H NMR and ^{13}C NMR of 4-Nitrocatechol.

Methylsuccinic acid (**5**): ^{1}H NMR (400 MHz, DMSO) δ = 12.14 (s, 2H), 2.66 (dqd, *J*=8.4, 7.2, 5.6, 1H), 2.55–2.44 (m, 1H), 2.29 (dd, *J*=16.7, 5.7, 1H), 1.09 (d, *J*=7.2, 3H). ^{13}C NMR (101 MHz, DMSO) δ = 176.48, 173.06, 37.24, 35.23, 16.88.

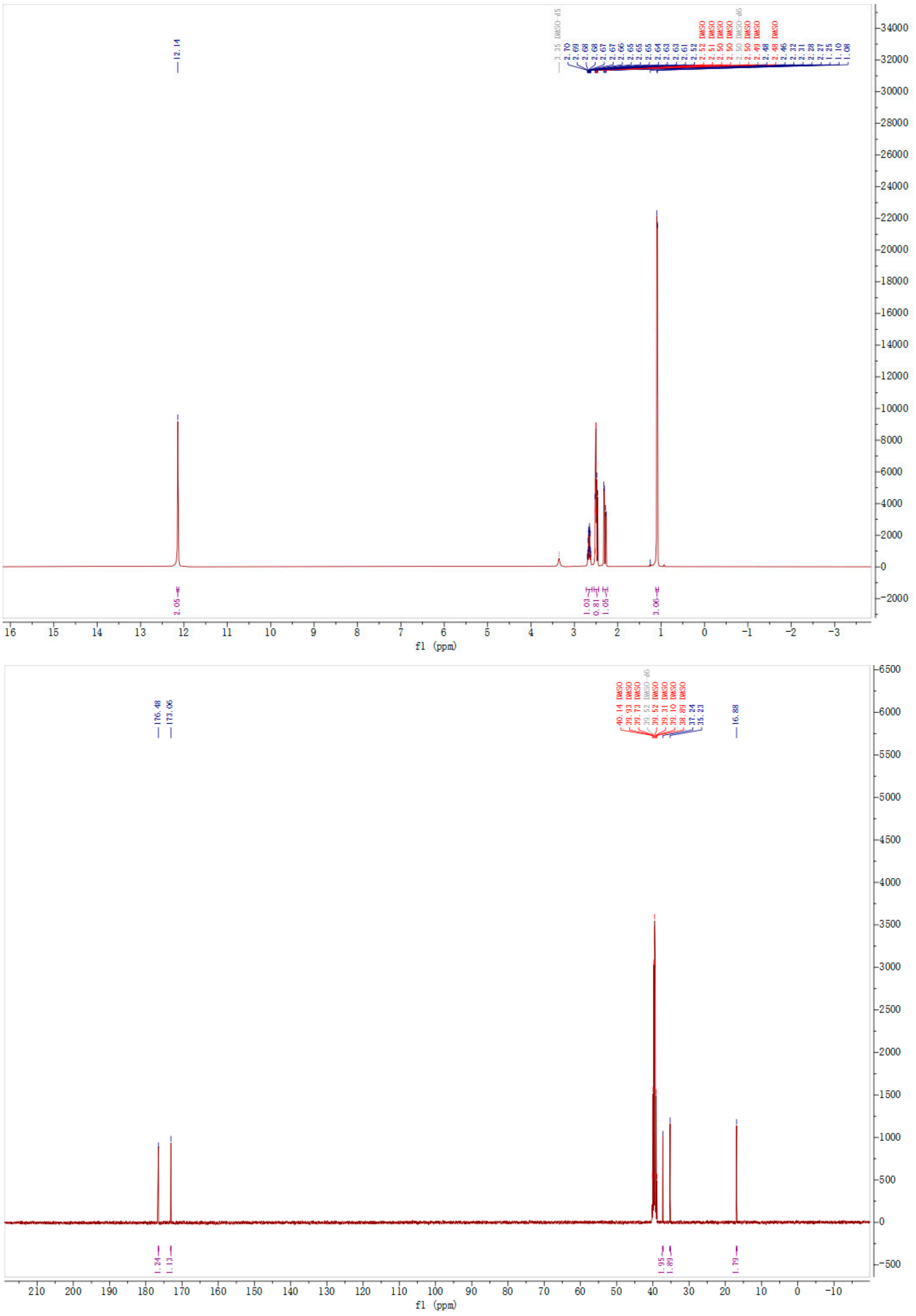

Figure S8 ^{1}H NMR and ^{13}C NMR of Methylsuccinic acid.

Phenyllactic acid (6): ^{1}H NMR (400 MHz, DMSO) δ = 7.29–7.16 (m, 5H), 5.33 (s, 1H), 4.15 (dd, *J*=8.3, 4.5, 1H), 2.97 (dd, *J*=13.8, 4.5, 1H), 2.78 (dd, *J*=13.8, 8.3, 1H). ^{13}C NMR (101 MHz, DMSO) δ = 175.18, 138.18, 129.44, 128.01, 126.15, 71.10, 40.05.

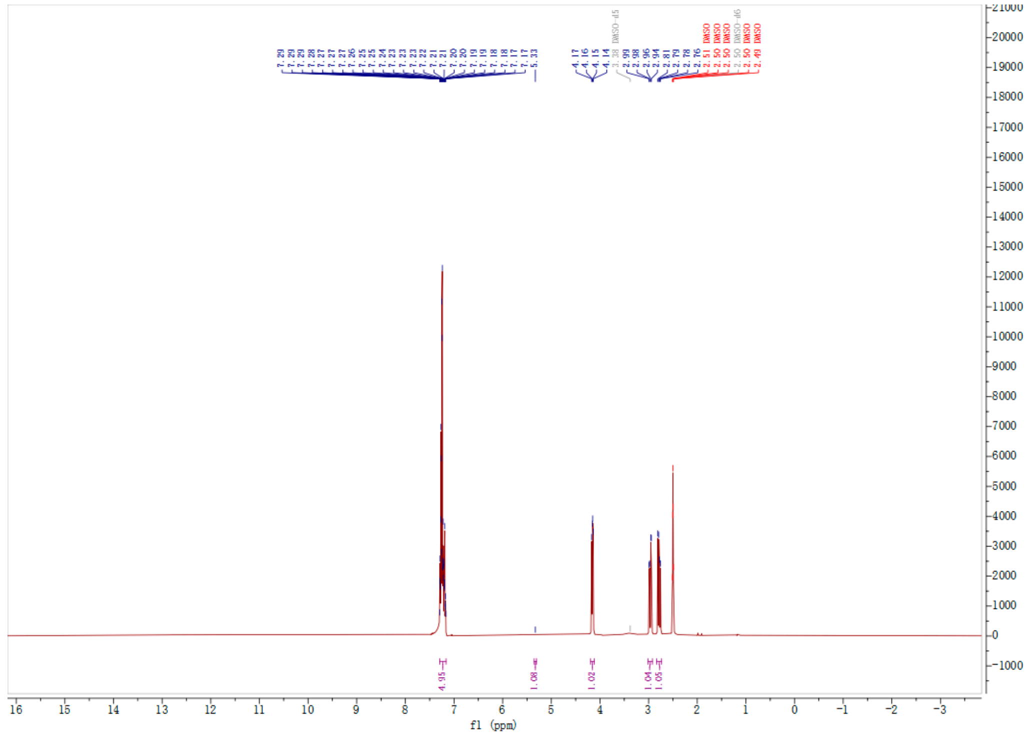

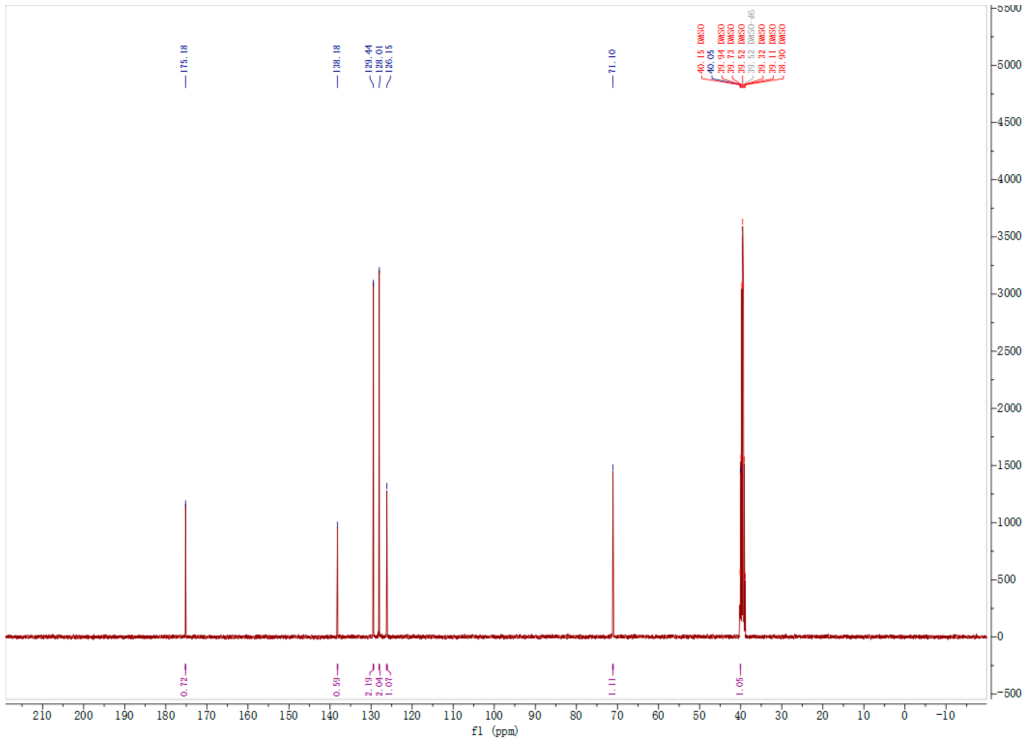

Figure S9 ^{1}H NMR and ^{13}C NMR of Phenyllactic acid.

5,6-Dimethylbenzimidazole (**7**): ^{1}H NMR (400 MHz, DMSO) δ = 12.15 (s, 1H), 8.04 (d, *J*=1.2, 1H), 7.39 (s, 1H), 7.28 (s, 1H), 2.30 (s, 6H). ^{13}C NMR (101 MHz, DMSO) δ = 140.93, 130.67, 115.57, 19.95.

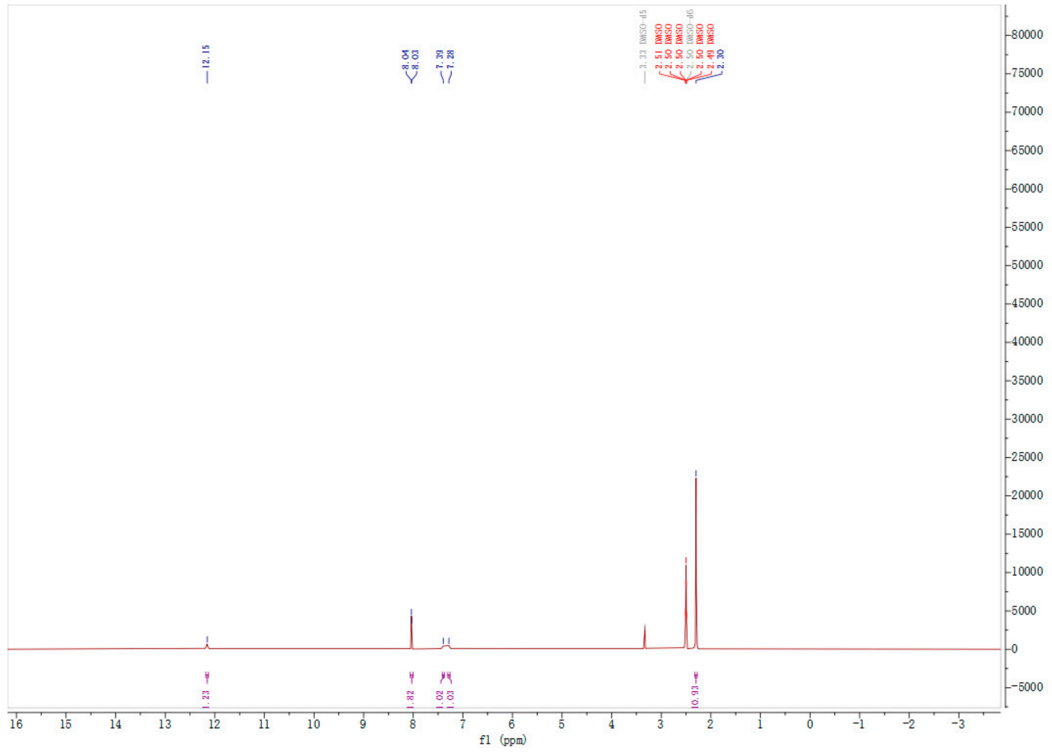

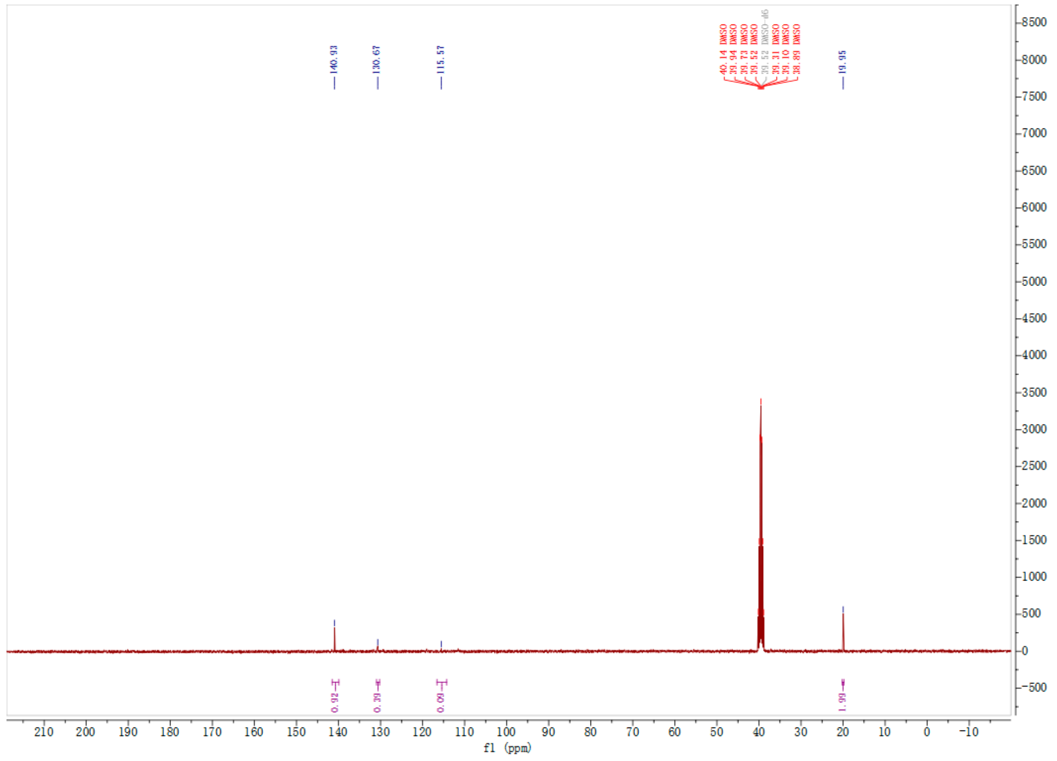

Figure S10 ^{1}H NMR and ^{13}C NMR of 5,6-Dimethylbenzimidazole.

Ishigamide (8): ^{1}H NMR (400 MHz, DMSO) δ = 7.17 (dd, *J*=12, 15, 1H), 6.60 (dd, *J*=10, 15, 1H), 6.40 (dd, *J*=11, 15, 1H), 6.33 (dd, *J*=11, 15, 1H), 6.25 (dd, *J*=12, 15, 1H), 6.15 (dd, *J*=11, 14, 1H), 5.97 (d, *J*=15.6, 1H), 5.85 (m, 1H), 3.72 (m, 1H), 3.48 (m, 2H), 2.16 (m, 2H), 1.54 (m, 2H), 1.46 (m, 2H), 1.14 (d, *J*=5.9, 3H). ^{13}C NMR (101 MHz, DMSO) δ = 178.25, 169.44, 139.53, 137.82, 136.57, 131.51, 130.42, 125.01, 65.18, 38.22, 34.22, 27.62, 22.92.

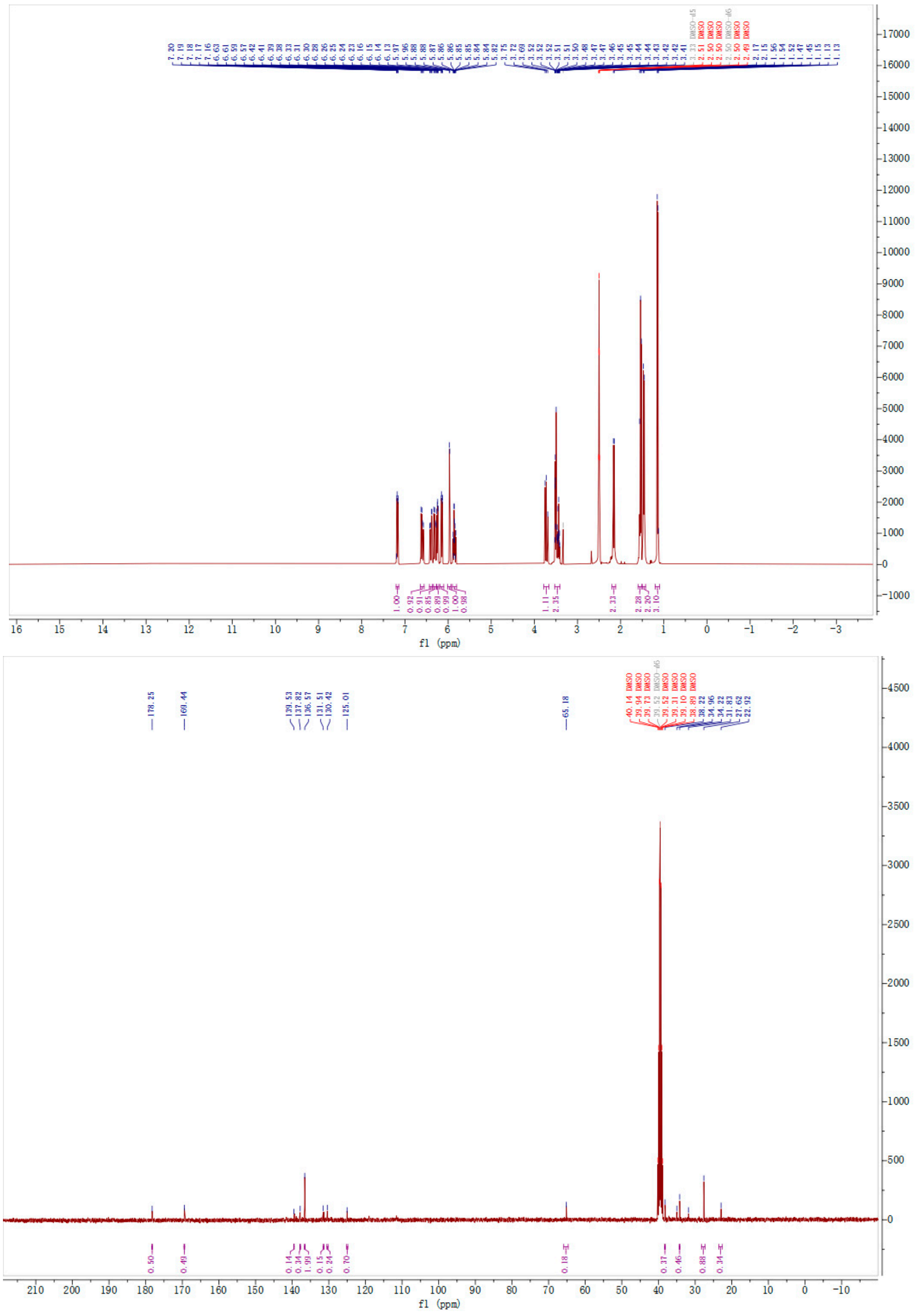

Figure S11 ^{1}H NMR and ^{13}C NMR of Ishigamide.

Table S4 The primer list for the RT-PCR.

Genes	Sense primer	Anti-sense primer	Product length (bp)
154	5'-GGTGACCTGGCATGTGATGGAGCG-3'	5'-CGTAGCAGGCGAGGTTGAGCGTGTA-3'	113
155	5'-GTGCCCACCCTGAACTACGAACGC-3'	5'-CCGATGCCGAACGAACTGACCC-3'	134
156	5'-GAGAACGGTCTGACGGGTCTGGA-3'	5'-AGGGCGATCTGTTCGGTGGG-3'	86
157	5'-GCTTCGACATCATCGCCAACCA-3'	5'-TACCGCCGCCACGAACTCCT-3'	110
158	5'-GGTACTTCCACCTGCTGACGCTCTG-3'	5'-TTGGTGTCGCCGTCGCTGTG-3'	139
159	5'-CAAGCACCTGAGCGAGGAGGACAC-3'	5'-TCGAGACGCATGGGCACCTGAAGC-3'	135
160	5'-ACGGCTTCGTGGACGGTGAGGGAGT-3'	5'-CGAGCCCTTCAGGACGGCATAGA-3'	101
457	5'-GCCAAGGTGAAGAAGGCGCTCAA-3'	5'-CGACTCCAGGGTCAGGTCGTACAGG-3'	123
466	5'-TGCGAGCTGATCGGGGTGG-3'	5'-GGCGAGGTAGCGGGTGAACG-3'	129
467	5'-TTCGTTGCCTGTTCACTGCTGG-3'	5'-AAGGCCATGTGGTGGCTGTTG-3'	101
468	5'-GTCTCGTCCGTCCCGAACTCCA-3'	5'-ACATCACCACCTCGCCCGTCCT-3'	91
469	5'-GTTCCACGCCTCCTACGACGACC-3'	5'-CGCAGCCGATCCGTGTCCAG-3'	93
470	5'-CGTTCACCCTGCGTTCCCTCG-3'	5'-ACTGCCATCCGCTCGCCCTC-3'	88
471	5'-CGTCTACGCCGTGCAGTTCCAG-3'	5'-ACCGCCTTGCCCGACTTCCT-3'	138
472	5'-CCCTCACCGTGAACGGGAAGCT-3'	5'-ATCTCGCCGAACAGCCCGCACAAG-3'	124
476	5'-CCCCGAGCATCTCCACTCCTCTT-3'	5'-GTTCCGCCCGCATCCATTTCT-3'	104
477	5'-CCTGGAGAAGCCGTGGGTCG-3'	5'-GCGGCGGGCAGACTGAGGTA-3'	120
479	5'-TCCAGCGGCTCCTTCCTCTTCT-3'	5'-CATCGGATCAGCGCCGTTTCT-3'	125
480	5'-TCAACGAGATCACCACCCGCTACC-3'	5'-CCTGCTGCGGTGAACTTCCCTGT-3'	147
584	5'-TTTTGTGGCGTGTCCGTTTGG-3'	5'-CGCACCTTCACCTGGTCATCG-3'	123
585	5'-GCGGAGCGGATCGCCTTCAT-3'	5'-CGCAATCGGTGCCCTCGGTA-3'	139
598	5'-CGGGGAGACGGGCGAACTGT-3'	5'-GCGGGGAGCCGAAGGGATT-3'	110
602	5'-CACGGCAATCTGCTGCACAACG-3'	5'-GGTGCGAGCGTCTGGAGGATGA-3'	125
605	5'-CGCGGGCACGGTCATACTC-3'	5'-GTGGCAGTGGGTGGACAGGAT-3'	121
652	5'-ACCGCCCGCCTCGTCCTCCTT-3'	5'-CCACGGCGTATCCGCTCCAGT-3'	89
654	5'-CCGACTCCGTCCGTCATCTGC-3'	5'-GGTGGTCATCCAGGGGAACAGG-3'	101
788	5'-CGGCATCAGCGTTTCGACCAT-3'	5'-GGCATCCGAGACGAGCAGGGTA-3'	87
801	5'-TGTTCGGCTGGTGGAAGTGGG-3'	5'-GCTTGGCGGAGACGATGGTGAG-3'	144
804	5'-GGCATCTACCGCCGCCTCCT-3'	5'-TCTCGTGGCCCGGCAGTGAG-3'	85
805	5'-ACTGGTCGCATACGAAACGGAACG-3'	5'-GGCCAGCAGCAGCTTCAGCCT-3'	97
1598	5'-CGAGATGCGGAAGCTGTGGACC-3'	5'-GCCCGGTGTTGACCGCGTAGAA-3'	86
1599	5'-GCCACCAGCATCGAAAGGGAGT-3'	5'-CCGGGACGAAGTAGTCGAAGAGGT-3'	106
1691	5'-TCGGCATCATCCACGACCTGA-3'	5'-CAGCCCGAAGTCCTCGTACAGC-3'	95
2647	5'-TTGCTGAGTGCCACGGACGAA-3'	5'-GGCCACGACGAGGGAAACCA-3'	81
2648	5'-ACAACGACCTGTGCTCACTTCCC-3'	5'-ACCGCCTCTTCGAGCGTCAGTC-3'	103
2649	5'-CGGACCACCTGTACGGCTACC-3'	5'-GGCGTGCCCTCTGCCATCT-3'	88
3832	5'-CAAGCGGGTCAACAGCAAGGTG-3'	5'-ACGGCATCGGCCAGTTCGTC-3'	108
3833	5'-GGCTCATGGGCGTCCAGGTCTA-3'	5'-TCCAGGATGCGGCGGGTGTT-3'	82
3837	5'-GACCTGCTTCGCCCTGACCG-3'	5'-CGTTGCCGACGTACCGCTTCT-3'	122
3838	5'-GCCGACGGGCAGGCTATTGT-3'	5'-TTCGCCCACCAACGCAACG-3'	114

3839	5'-ACGACATGCTGGCGAAGGACC-3'	5'-CGACGAGATGCCGAGGACGA-3'	128
3842	5'-ATCGCCCGTCCGCCATCAGT-3'	5'-CGGTCATCTCCTCGAAATCCACCA-3'	135
3844	5'-GCCGACGGCACGATGGAGAT-3'	5'-AAGAAGGCCACGAAGCGGGCGAGA-3'	95
3845	5'-ACGCTCCAGTTCGTCGCGGACAT-3'	5'-GGTTCACCGTTCTTGCGGTTGC-3'	146
3850	5'-CGTCGCCGTGAAGTCGATGCT-3'	5'-TGGTTGATGGTGGGCGGGAT-3'	111
3851	5'-GGCGAGGGCAACAGGACGGT-3'	5'-GCGTGCCTGGGTGTACGACTGG-3'	117
3852	5'-GCGGGCACCATCGTCAACCT-3'	5'-CACTCCTTGGCGAGGGACTTGG-3'	119
3853	5'-TGGTGGCACGACAGCGACGACA-3'	5'-CCTTGATGGAGGCGAAGGAGAACG-3'	118
3854	5'-GCGAAGGACAGACGGTCAACCC-3'	5'-GGAAGCTGGAGATCCACTTGATGC-3'	135
6001	5'-GGAGTCCCTGATCCCGCTGCAC-3'	5'-GCCCCGTGATAGGCGAGAAAGG-3'	138
6003	5'-CATCAACGAGCCCGACCCC-3'	5'-CGACCTCCATGACGTGCAGGTAC-3'	92
6004	5'-TACAAGCGGTCGCTCCAGATGC-3'	5'-AACAGGCGGATGCCGTGGTC-3'	89
6005	5'-GGCCGAACTGACCGACGACA-3'	5'-GACGGGCGGGTTGAAGACA-3'	139
6006	5'-CGAGCACAACGGCACCTTCC-3'	5'-GCCGAACCGTCGGTCCAGTA-3'	87
6009	5'-GTGAGCGACGTGAGCGAAGTGAG-3'	5'-CGATGGCGGCGAAGTGCTG-3'	100
6480	5'-TCCACATGGCGTGCCAGAGC-3'	5'-TGCCGTCGAAGGTGAGGTTCAG-3'	87
6481	5'-CGCTCCAGCTCGGTTCGTTGA-3'	5'-TGCGGCGTCGGCTCATTCA-3'	139
6482	5'-CCATTCGCCTTTGGTGGAGCC-3'	5'-ACCACGGGGATGGACGGCTC-3'	87
6483	5'-AGGGCTGCGGCTCGTCTCGT-3'	5'-CAGCACGTCCCGGAAGTTGACA-3'	103
6484	5'-GGTCAAGTCCAACATCGGCCACA-3'	5'-TGGGCTCGTCCACGTTCAGGGT-3'	116
6485	5'-CCTGGAGACCTCCTGGGAGACCTT-3'	5'-CGCCGTTGACGCCGATGAA-3'	95
7016	5'-CATGGGTGGAACTGATGGGAAGG-3'	5'-CCGCCGAGAACCGTGGTGATA-3'	91
7023	5'-CCGCCCTCCTCCAGCACTTC-3'	5'-CCATCAGGTCCGCGAGACGC-3'	81
7025	5'-CTCGACCACATCAGCGCCTGGGAGT-3'	5'-CGAAGAAGCTGCGGGAGAACACC-3'	97
7027	5'-GCATCCGCACCGCCTTCGT-3'	5'-AGACCGTCGCACGCCACCAC-3'	127
7028	5'-GGCGATGGAAGCCGAGGTGG-3'	5'-GGAGTCGAAGTTGGCGTCGATGAA-3'	130
7715	5'-CTCGCCTGCCTCAAGCTCTTTCT-3'	5'-GGTGATGGTCCGTACTGCCTGGTC-3'	147

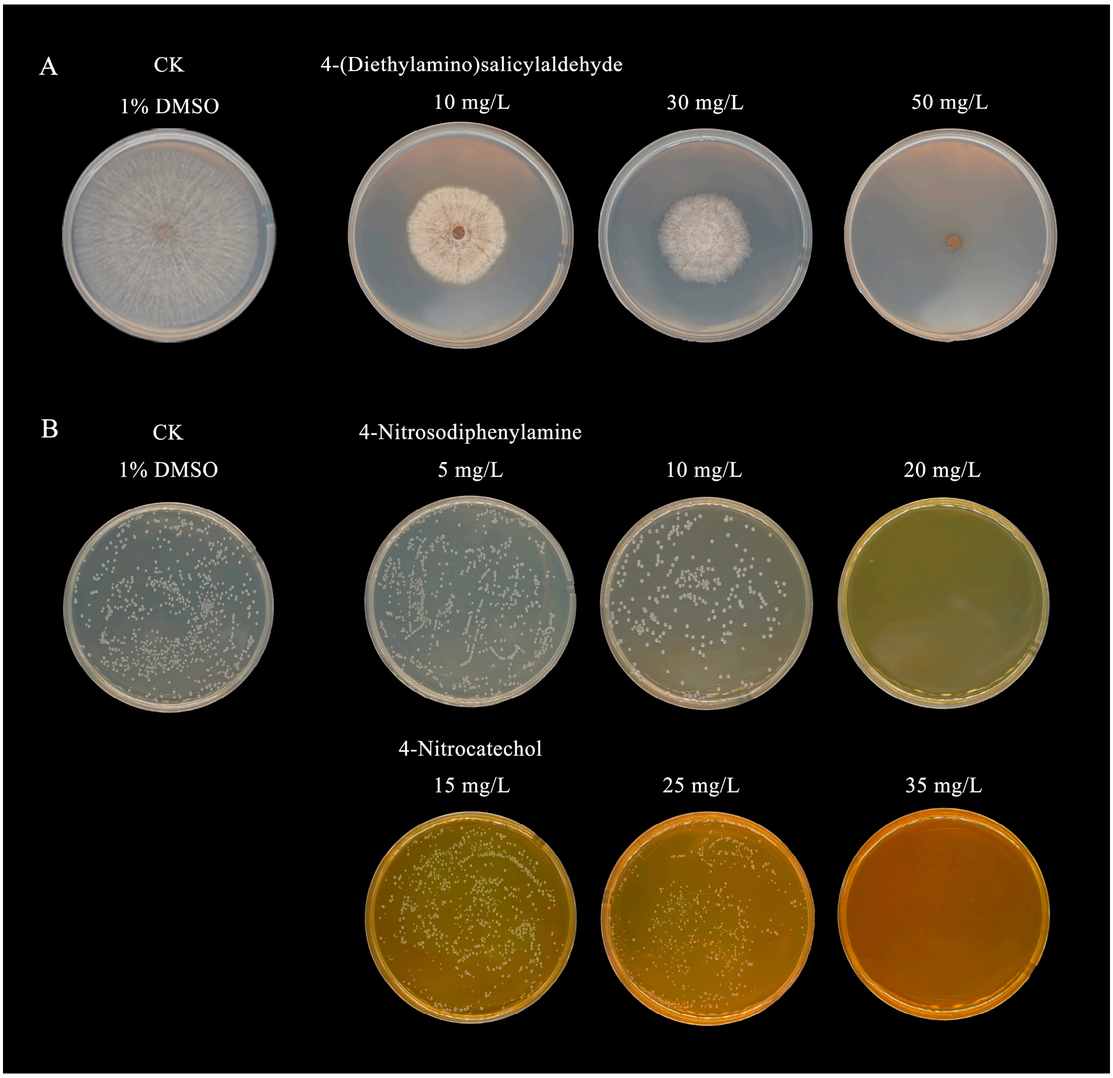

Figure S12 Inhibitory effect of highly active substances. (A) The inhibitory effect of 4-(Diethylamino)salicylaldehyde on *R. solani*. (B) The inhibitory effect of 4-Nitrosodiphenylamine and 4-Nitrocatechol on *E. amylovora*.
